# Advanced Manufacturing Technologies of Thermoplastic Composites

**DOI:** 10.3390/ma17225564

**Published:** 2024-11-14

**Authors:** Tian Zhao

**Affiliations:** Institute of Advanced Structure Technology, Beijing Institute of Technology, Beijing 100081, China; t.zhao@bit.edu.cn

Thermoplastic composites are becoming increasingly attractive to the aerospace and automotive industries owing to their outstanding mechanical properties and cost-effective manufacturing processes. Some advanced manufacturing techniques, e.g., 3D printing, filament winding and welding, are particularly well-suited for this type of composites. However, high temperatures and pressures are normally required during the fabrication due to the intrinsic properties of thermoplastic resin, e.g., a relatively high Tg and viscosity, which introduce additional difficulties and challenges for the manufacturing process. Numerous researchers have worked to gain insights into the mechanisms that underlie different manufacturing processes of thermoplastic composites in order to promote their applications in engineering structures.

This Special Issue is titled “Advanced Manufacturing Technologies of Thermoplastic Composites” and is dedicated to providing readers with an overview of the current research progress on different manufacturing techniques of thermoplastic composites. The Special Issue includes ten high-quality research articles, the collection of which covers a period of almost two years, while more than 60 authors from different institutions participated to contribute their scientific outputs.

Geng et al. [1] investigated the influence of process parameters on the mechanical properties of thermoplastic composite materials (CFRPs) using laser-assisted CF/PPS winding forming technology. A numerical model was established to simulate the heat transfer that occurs during the winding process, as well as the tensile behavior of the composite specimens. Yu et al. [2] analyzed the effects of surface treatment on the resistance welding process for unidirectional carbon fiber/PPS composites. A quantitative processing window was proposed for resistance welding to achieve the best weld strength. Yao et al. [3] reported research on the interfacial enhancement of CF/PA6T composites with pre ultrafine PA6T powder as an emulsion sizing agent. It was found that the mechanical performance, including the tensile, shear and interlaminar strength, was obviously improved for the processed composites. Li et al. [4] optimized the formulation design and foaming processes and achieved mechanical property enhancement using a carbon-fiber-reinforced PVC composite foam (CF/PVC). The results indicated a reduction in VOC emission in automotive interior leather applications. The influence of thermal parameters on the self-nucleation behavior of a PPS during secondary thermoforming was systematically studied by Ren et al. [5]. Different mechanisms were found for the two thermal cycles, while it was shown that they will both generate self-nucleation behavior. To achieve efficient and strong connections between the metal and polymer components, Zhou et al. [6] used a hot pressure-welding technique to join AA6061 and CF/PA66 composites and found that the aluminum alloy’s surface morphology has the greatest impact on the mechanical property of the welded joint. Wu et al. [7] quantitatively investigated the typical manufacturing defects, e.g., fiber bundle bias distribution and void contents, in 3D-printed CF/PLA specimens using a micro-CT technique. Wang et al. [8] performed a detailed analysis of prepreg trajectories in relation to the shell geometry, accompanied by an in-depth investigation of the underlying causes of wrinkling on dome surfaces. Zhao et al. [9] explored the possibility of combining the compression-molding of CCF-PAEK and injection of SCF-PEEK. The interfacial behavior was significantly affected by the processing temperature. A parametric study, considering the lap length, adhesive layer thickness, adhesive layer shape, adhesive layer overflow length and laminate lay-up, was conducted by Chen et al. [10] to characterize the shear strength of adhesively bonded composite joints by using both numerical and experimental methods.

As is known, the “advanced manufacturing technologies of thermoplastic composites” is a comprehensive topic, and therefore, it is not possible to include research on all aspects of this research area. However, we believe that the collected papers provide a further understanding of the physical mechanisms of different manufacturing technologies and the mechanical behavior of thermoplastic composites. We hope that further progress can be achieved based on the ideas introduced in this Special Issue.
